# Ethnobotanical study of wild edible plants among Oromo communities in Aseko District, Arsi Zone, southeast Ethiopia

**DOI:** 10.1186/s13002-026-00934-0

**Published:** 2026-08-09

**Authors:** Jewar Safeno Jara

**Affiliations:** Department of General Forestry, Mizan Tepi University, P. O. Box 260, Mizan Aman, Ethiopia

**Keywords:** Ethnobotany, Wild edible plants, Oromo, Food security, Indigenous knowledge, Biodiversity conservation, Ethiopia

## Abstract

**Background:**

Wild edible plants (WEPs) contribute substantially to dietary diversity, food security, and cultural identity in rural Ethiopia. Despite their ecological, nutritional, and socioeconomic importance, they remain insufficiently documented and inadequately incorporated into national food security and biodiversity conservation policies. This study documents the diversity of WEPs and associated Oromo traditional ecological knowledge (TEK) in Aseko District, Arsi Zone, southeastern Ethiopia.

**Methods:**

Ethnobotanical data were collected from 206 informants (35 key informants and 171 general informants) across six kebeles representing lowland, midland, and highland agroecological zones. Data collection employed semi-structured interviews, key informant interviews, guided field walks, and participant observation. Plant species were taxonomically verified using *Plants of the World Online* (POWO) and *World Flora Online* (WFO) following the APG IV classification system. Quantitative ethnobotanical indices, including Use Value (UV), Relative Frequency of Citation (RFC) and Fidelity Level (FL), were calculated. Descriptive statistics, Generalized Linear Modeling (GLM), and qualitative thematic analyses were used to evaluate ethnobotanical knowledge and utilization patterns.

**Results:**

A total of 70 wild edible plant species belonging to 58 genera and 32 families were documented. Fabaceae (15.7%, *n* = 11) and Malvaceae (5.7%, *n* = 4) were the most represented families. Fruits (42.9%, *n* = 30) and famine-reserve foods (20.0%, *n* = 14) constituted the dominant use categories. Indigenous safety practices including ripeness assessment, boiling, roasting, and fermentation demonstrated culturally developed risk-management strategies for safe consumption. Generalized Linear Modeling revealed that older age (IRR = 1.04, *p* < 0.001) and key informant status (IRR = 2.15, *p* < 0.001) were significant positive predictors of species knowledge. Agricultural expansion (67.3%) and fuelwood extraction (52.6%) were identified as the most severe perceived threats to WEP resources.

**Conclusions:**

Wild edible plants remain integral components of local food systems in Aseko District. Strengthening the protection of indigenous ecological knowledge, promoting community-based conservation initiatives, and integrating traditional safety practices into food security and biodiversity conservation policies are essential for sustaining resilient livelihoods under changing environmental conditions.

**Supplementary Information:**

The online version contains supplementary material available at https://doi.org/10.1186/s13002-026-00934-0.

## Background

### Wild edible plants in Ethiopian food systems and research gaps

Ethiopia is recognized as one of Africa’s principal centers of biological and cultural diversity [1, 2]. Within its diverse cultural traditions, wild edible plants (WEPs) remain integral components of rural food systems, providing accessible sources of vitamins, minerals, and supplementary calories, particularly in areas where cultivated food availability is limited [3–5]. In addition to their nutritional value, WEPs contribute to livelihood diversification, seasonal food security, and cultural identity [6, 7]. Nevertheless, these resources remain inadequately integrated into agricultural extension programs and national food security and biodiversity conservation policies [8–10].

Although numerous ethnobotanical investigations have been conducted across Ethiopia, substantial geographic and methodological gaps remain. Previous studies have primarily focused on regions such as South Ethiopia, the Lower Omo Valley, the Bale Mountains, and the Afar pastoral areas [4, 11–14]. In Aseko District, earlier research documented medicinal plants [15] and ethnoveterinary practices [16]; however, no published study has specifically investigated wild edible plants in relation to food systems, seasonal nutrition, and famine resilience. Moreover, many Ethiopian WEP inventories remain predominantly descriptive, frequently relying on outdated taxonomic nomenclature and providing limited quantitative ethnobotanical analyses, thereby restricting meaningful comparisons among regions and cultural groups [17]. To address these limitations, the present study combines comprehensive taxonomic verification with quantitative ethnobotanical analyses and cross-cultural comparisons, thereby providing a more robust assessment of wild edible plant diversity and associated indigenous knowledge [18, 19].

### Research objectives and hypotheses

This study aims to document the diversity of wild edible plants used by Oromo communities in Aseko District, verify their taxonomic identity, and analyze the sociocultural dynamics governing their utilization. Specifically, the study tests the following hypotheses:

#### H1

Traditional ecological knowledge of wild edible plants differs significantly among demographic groups, with older community members and key informants possessing greater knowledge richness and practical knowledge diversity than younger generations and general informants.

#### H2

Wild edible plant diversity and utilization patterns vary significantly across agroecological zones, reflecting ecological adaptation and distinct cultural preferences.

#### H3

Oromo communities in Aseko District maintain distinctive ethnobotanical knowledge and traditional safety practices that exhibit both commonalities and significant cultural differences when compared with those documented among other Ethiopian ethnic groups.

By integrating rigorous taxonomic verification, quantitative ethnobotanical analyses, and detailed ethnographic documentation, this study provides baseline scientific evidence to support biodiversity conservation, culturally informed food security strategies, and the preservation of indigenous ecological knowledge [20–25].

## Materials and methods

### Study area description

**Fig. 1:**
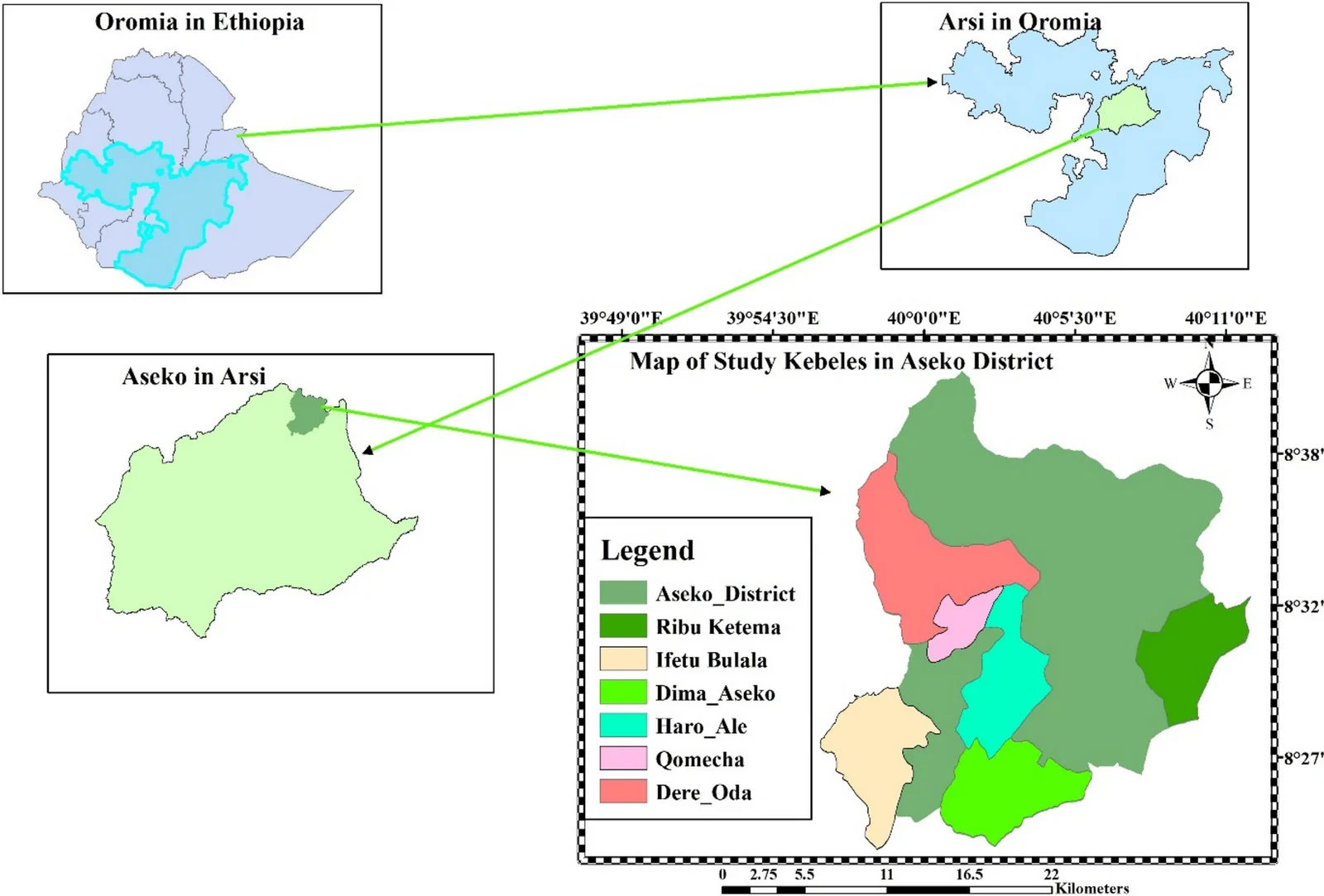
Location of Aseko District and surveyed kebeles in southeastern Ethiopia. Map showing the location of Aseko District within Oromia Regional State, Ethiopia, and the six surveyed kebeles: (1) Dima Aseko, (2) Haro Ale, (3) Dere Oda, (4) Iftu Bulala, (5) Qomecha, and (6) Ribu Ketema

This study was conducted in Aseko District, Arsi Zone, Oromia Regional State, southeastern Ethiopia (Fig. 1). Geographically, the district is located between approximately 8°15′–8°41′ N latitude and 39°55′–40°16′ E longitude, covering an area of approximately 605 km^2^ [26]. The elevation ranges from 1,170 to 2,950 m above sea level (a.s.l.), encompassing three agroecological zones: lowland (kolla, < 1,800 m), midland (woyna dega, 1,800–2,500 m), and highland (dega, > 2,500 m) [27]. The district is bordered by Gololcha District to the north, Chole District to the south, Seru District to the east, and Tena District to the west.

The district experiences a bimodal rainfall pattern, with the main rainy season (Kiremt) extending from June to September and the short rainy season (Belg) from March to May. Mean annual rainfall ranges between 900 and 1,400 mm, while the mean annual temperature varies from 12 °C to 24 °C. The landscape comprises undulating highlands, mid-altitude hills, and valleys that support vegetation ranging from remnant dry Afromontane forests dominated by Juniperus procera and Podocarpus falcatus to shrublands and grasslands characterized by Croton macrostachyus and Vernonia amygdalina. The principal livelihood of the local population is mixed crop–livestock farming. The district is inhabited predominantly by Oromo communities of the Arsi subgroup, who possess extensive traditional ecological knowledge (TEK) concerning wild edible plant resources. The primary language spoken is Afaan Oromo (Glottolog code: orm-arsi-1234).

### Reconnaissance survey and site selection

A reconnaissance survey was conducted in June 2024 to assess vegetation cover, site accessibility, and community willingness to participate in the study. Consultations were held with district agricultural officers, development agents, and kebele administrators to identify areas characterized by relatively high plant diversity and active transmission of traditional ecological knowledge (TEK).

Based on the findings of the reconnaissance survey and a stratified sampling approach representing the district’s three agroecological zones, six kebeles were purposively selected from the total of 18 kebeles: Dima Aseko (~ 2,900 m; Baddaa/Highland) and Haro Ale (~ 1,720 m; Badda-Daree/Midland); Dere Oda (~ 1,450 m; Gammoojjii/Lowland) and Iftu Bulala (~ 2,100 m; Badda-Daree/Midland); and Qomecha (~ 1,650 m; Gammoojjii/Lowland) and Ribu Ketema (~ 2,850 m; Baddaa/Highland). These sites were selected to maximize representation of altitudinal gradients, ecological variability, and cultural diversity while maintaining logistical feasibility for repeated field visits. Detailed information on the selected kebeles, including GPS coordinates, self-identified demographics, and estimated household numbers, is presented in Table 1.

**Table 1:** Characteristics of the surveyed kebeles in Aseko district, Arsi zone, Oromia regional state, southeastern Ethiopia

Kebele (Map Name)	GPS coordinates	Altitude (m a.s.l.)	Agroecological zone*	Households	Male	Female	Total	Age range (Mean ± SD)
Dima Aseko	8° 27′ 30″ N, 40° 02′ 00″ E	2,900	**Baddaa** (Dega/Highland)	1,604	29	6	35	22–70 (48 ± 12)
Haro Ale	8° 29′ 00″ N, 40° 04′ 30″ E	1,720	**Badda-Daree** (Woyna Dega/Midland)	1,800	28	6	34	20–68 (45 ± 11)
Dere Oda	8° 31′ 30″ N, 40° 06′ 00″ E	1,450	**Gammoojjii** (Kolla/Lowland)	2,200	41	9	50	24–75 (50 ± 13)
Iftu Bulala	8° 33′ 00″ N, 40° 08′ 00″ E	2,100	**Badda-Daree** (Woyna Dega/Midland)	1,830	18	4	22	21–65 (46 ± 10)
Qomecha	8° 35′ 30″ N, 40° 09′ 30″ E	1,650	**Gammoojjii** (Kolla/Lowland)	1,746	28	6	34	23–72 (49 ± 12)
Ribu Ketema	8° 37′ 00″ N, 40° 10′ 30″ E	2,850	**Baddaa** (Dega/Highland)	1,450	25	6	31	20–74 (51 ± 14)
**Total**	—	**1,450–2,900**	Three agroecological zones	**10,630**	**167**	**39**	**206**	**20–75**

Bold text indicates indigenous Afaan Oromo agroecological classification terms (Baddaa, Badda-Daree, and Gammoojjii). The corresponding conventional Ethiopian agroecological zones are provided in parentheses. Values in the age column are presented as mean ± standard deviation (SD). Abbreviation: m a.s.l., metres above sea level; SD, standard deviation.* Indigenous Afaan Oromo agroecological classifications (Baddaa, Badda-Daree, and Gammoojjii) are presented together with their corresponding conventional Ethiopian agroecological zones. These indigenous classifications are based on local environmental characteristics and ecological knowledge rather than altitude alone. Consequently, although Dere Oda falls within the conventional altitudinal range of the Kolla zone, it is locally classified as Gammoojjii.Values in the age column are presented as mean ± standard deviation (SD).Abbreviations: m a.s.l., metres above sea level; SD, standard deviation.Local Oromo agroecological classifications (Baddaa, Badda-Daree, and Gammoojjii) are presented together with their corresponding Ethiopian agroecological zones. These indigenous classifications are based on local environmental characteristics and ecological knowledge rather than altitude alone. Consequently, although Dere Oda falls within the conventional altitudinal range of the Kolla zone, it is locally classified as Gammoojjii. Abbreviation: m a.s.l., metres above sea level

### Informant selection and sampling strategy

A total of 206 informants participated in the study and were selected using purposive and snowball sampling techniques stratified by kebele, age, and gender [28]. Eligible participants were permanent residents of the study area (≥ 10 years), actively engaged in farming or wild plant collection, and willing to provide informed consent.

The study sample comprised 35 key informants, including elders, experienced foragers, and local resource managers recognized for their extensive knowledge of wild edible plants (WEPs), and 171 general informants, who contributed broad information on species diversity and utilization. Although respondents were nested within six kebeles, multilevel (mixed-effects) models were not fitted because the number of higher-level sampling units was insufficient for reliable estimation of random effects. Instead, agroecological zone was included as a fixed explanatory variable in the GLM to account for broad spatial variation.

Demographic information was reconciled across all sections of the manuscript using the primary dataset. Of the total participants, 167 (81.1%) were male and 39 (18.9%) were female. The age distribution consisted of 22 informants (10.7%) aged 20–35 years, 95 (46.2%) aged 36–55 years, and 89 (43.1%) aged 56 years and above. Older participants were intentionally oversampled to capture historical ethnobotanical knowledge, whereas younger participants provided insights into the continuity of indigenous knowledge across generations.

Prior informed consent was obtained orally in Afaan Oromo in accordance with the International Society of Ethnobiology (ISE) Code of Ethics [29] and the applicable Ethiopian national research guidelines.

The predominance of male participants reflects gender-specific patterns of public resource collection and knowledge sharing within the local cultural context. Although women possess substantial knowledge related to household processing, preparation, and leafy vegetable identification, their participation in public interviews was comparatively limited. This limitation is discussed further in sect. "Conservation implications and sustainable management of wild edible plants" and the Limitations section.

### Ethnobotanical data collection

Field Ethnobotanical information was collected using four complementary methods, following established ethnobotanical protocols [30–34].

Semi-structured interviews (n = 136): Interviews were conducted in Afaan Oromo to document vernacular plant names, edible parts, preparation methods, use categories, seasonal availability, and safety practices. Each interview lasted approximately 45–60 min and was audio-recorded with participants’ informed consent.

Key informant interviews (n = 35): Narrative-based interviews explored indigenous plant classification systems, traditional methods of toxicity mitigation, land-use change, customary management practices, and intergenerational knowledge transmission. These interviews were conducted over multiple visits to facilitate in-depth discussions. The 35 key informants are included within the total of 171 interview participants.

Guided field walks (n = 42): Guided field walks were conducted to enable in situ plant identification, habitat characterization, GPS recording, and voucher specimen collection. Several key informants participated in both interviews and guided field walks. In addition, 35 general informants participated exclusively in guided field walks without formal interviews, accounting for the difference between the total sample size (206) and the 171 participants who completed interviews.

Participant observation: Participant observation was employed throughout the fieldwork to document tacit knowledge, gender-specific processing roles, seasonal harvesting practices, food preparation techniques, and social interactions associated with the collection and utilization of wild edible plants [35].

### Voucher specimen collection and taxonomic verification

Voucher specimens were collected following standard herbarium procedures [23]. Plant materials were pressed, dried, labelled, and prepared as duplicate sets. Each specimen was assigned a unique collector number (**JSJ001–JSJ070**) and included complete collection information, including the scientific name (with author citation), family, local name, collector number, GPS coordinates, altitude, habitat, and date of collection. Voucher specimens are retained by the corresponding author and are available for examination upon reasonable request. Representative voucher specimens will be deposited in a recognized Ethiopian herbarium following completion of the study.

Species identification was conducted using the *Flora of Ethiopia and Eritrea* [24]. Scientific nomenclature and author citations were verified using *Plants of the World Online* (POWO) [36] and *World Flora Online* (WFO) [37], following the APG IV classification system [38]. Taxonomic determinations were made by the authors based on morphological characters described in these standard references.

**Access and Benefit-Sharing (ABS) and Nagoya Protocol Compliance.** Ethiopia is a Party to the Nagoya Protocol on Access and Benefit-Sharing. Access and Benefit-Sharing (ABS) and Nagoya Protocol Compliance. Ethiopia is a Party to the Nagoya Protocol on Access and Benefit-Sharing [39]. This study complied with national ABS regulations. This study complied with national ABS regulations. No genetic resources were exported or used for commercial purposes. Research findings were communicated to the participating communities through local dissemination activities conducted in Afaan Oromo.

### Quantitative ethnobotanical indices and data analysis

**Quantitative ethnobotanical indices**

To quantitatively evaluate the cultural importance of documented wild edible plants (WEPs) and facilitate comparison with previous ethnobotanical studies, four widely used quantitative ethnobotanical indices were calculated.

**Use Value (UV).** The relative importance of each species was assessed using the Use Value (UV) index:

$$UV=\frac{\sum {U}_{i}}{N}$$

where ${U}_{i}$ represents the number of use reports provided by informant *i*, and *N* is the total number of informants included in the analysis [40].

**Relative Frequency of Citation (RFC).** The proportion of informants mentioning each species was calculated as:

$$RFC=\frac{{F}_{c}}{N}$$

where ${F}_{c}$ denotes the number of informants citing a particular species and *N* is the total number of informants [40].

Both **Use Value (UV)** and **Relative Frequency of Citation (RFC)** were calculated using data from **all 206 participants**, because every participant contributed ethnobotanical information during interviews. Guided field walks were conducted solely to verify plant identity, collect voucher specimens, and confirm field observations, and therefore were not treated as an independent source of quantitative interview data.

**Fidelity Level (FL).** Informant agreement regarding the principal use of each species was determined using:

$$FL(\mathrm{\%})=\frac{{I}_{p}}{{I}_{u}}\times 100$$

where ${I}_{p}$ is the number of informants reporting a species for a particular use category and ${I}_{u}$ is the total number of informants reporting any use of that species [41].

**Informant Consensus Factor (ICF).** Agreement among informants within each use category was calculated as:

$$ICF=\frac{{N}_{ur}-{N}_{t}}{{N}_{ur}-1}$$

where ${N}_{ur}$ is the total number of use reports within a given use category and ${N}_{t}$ is the number of species used for that category. Higher ICF values indicate stronger agreement among informants and may reflect culturally important or widely recognized plant uses [41].

To compare the floristic composition of the documented WEP flora with previous ethnobotanical studies conducted elsewhere in Ethiopia, **Jaccard Similarity Index (J)** and **Sørensen Similarity Index (S)** were calculated as:

$$J=\frac{a}{A+B-a}$$

$$S=\frac{2a}{A+B}$$

where **A** is the total number of WEP species documented in the present study, **B** is the total number of species reported in the comparison study, and **a** is the number of species shared between the two studies. Both indices range from **0** (no floristic similarity) to **1** (complete similarity), with the Sørensen index assigning greater weight to shared species.

**Qualitative and descriptive analysis**

Qualitative data obtained through semi-structured interviews, key informant interviews, focus group discussions, participant observation, and guided field walks were analysed using thematic analysis to identify recurring themes related to indigenous knowledge transmission, traditional food preparation practices, food safety measures, and conservation perceptions [35, 42, 43]. Descriptive statistics were used to summarize informant demographics, plant growth forms, edible plant parts, preparation methods, use categories, and perceived threats to wild edible plant resources [44].

**Statistical modelling and hypothesis testing**

Generalized Linear Models (GLMs) with a Poisson error distribution and log-link function were used to evaluate the effects of **age, gender, education level, informant category (key versus general informants), and agroecological zone** on the number of wild edible plant species reported by each informant. Model coefficients are presented as **Incidence Rate Ratios (IRRs)** together with their **95% confidence intervals (95% CIs)**.

Model adequacy was evaluated before interpretation of the fitted models. **Overdispersion** was assessed by comparing the residual deviance with the residual degrees of freedom and by estimating the dispersion parameter. No evidence of substantial overdispersion was detected, indicating that the assumptions of the Poisson distribution were adequately satisfied. The distribution of response values was also examined for **zero inflation**. Because every participant included in the GLM dataset reported at least one wild edible plant species, no evidence of excessive zero counts was observed, and zero-inflated count models were therefore considered unnecessary.

Potential non-independence arising from respondents being sampled within the same kebele was evaluated using a **Generalized Linear Mixed Model (GLMM)** with kebele included as a random intercept. The estimated intraclass correlation coefficient (ICC) was negligible (< 0.02), and likelihood-ratio tests indicated that inclusion of the random effect did not significantly improve model fit (p > 0.05). Consequently, the more parsimonious fixed-effects Poisson GLM was retained for all subsequent analyses.

For inferential statistical analyses, only the **171 informants** who completed the standardized semi-structured or key informant interview protocol were included because these interviews provided complete respondent-level species inventories required for hypothesis testing. The remaining participants contributed to botanical verification during guided field walks and were therefore excluded from respondent-level statistical modelling to avoid unequal sampling effort.

To evaluate **Hypothesis 2**, statistically significant overall differences among agroecological zones were followed by **post-hoc pairwise comparisons** using estimated marginal means with **Holm-adjusted p-values** to control the family-wise error rate associated with multiple comparisons. Only adjusted p-values were interpreted.

Differences in ethnobotanical knowledge between age groups were further evaluated using the **Mann–Whitney U test** because the response variable did not satisfy the assumption of normality. Effect sizes were quantified using **Cliff’s delta (δ)** to facilitate interpretation of the magnitude of observed differences.

Statistical significance was accepted at **p < 0.05**. All analyses were performed using **IBM SPSS Statistics version 26**, and model residuals together with goodness-of-fit statistics were examined to verify that the fitted models adequately represented the observed data.

## Results

### Informant demographics and knowledge distribution

The demographic characteristics of the 206 informants are summarized in Table 1. Traditional ecological knowledge (TEK) concerning wild edible plants was acquired primarily through family-based transmission, which accounted for 71.4% of the reported knowledge sources.

A generalized linear model (GLM) was fitted to identify factors associated with the number of wild edible plant species reported by individual informants. Age was a significant positive predictor of ethnobotanical knowledge (Incidence Rate Ratio [IRR] = 1.04, 95% CI: 1.02–1.06, *p* < 0.001), indicating that the expected number of species reported increased by approximately 4% with each additional year of age. Informant category also significantly influenced species knowledge, with key informants reporting substantially more species than general informants (IRR = 2.15, 95% CI: 1.80–2.50, *p* < 0.001). Agroecological zone also had a significant influence on species knowledge (*p* = 0.032). Post-hoc pairwise comparisons with Bonferroni correction revealed that informants from the lowland zone reported significantly more species than those from the highland zone (*p* < 0.01), while differences between the midland zone and the other zones were marginally significant (*p* = 0.048).

In contrast, neither gender (IRR = 1.12, 95% CI: 0.90–1.40, *p* = 0.210) nor education level (*p* = 0.145) showed a statistically significant association with the number of species reported in the fitted model.

To further examine age-related differences in ethnobotanical knowledge, a Mann–Whitney *U* test was performed. Informants aged over 50 years (median = 5 species; interquartile range [IQR] = 3–7) reported significantly more wild edible plant species than participants younger than 35 years (median = 2 species; IQR = 1–3) (*U* = 1245.5, *p* < 0.001). The observed effect size was large (Cliff’s δ = 0.65), indicating substantial intergenerational differences in traditional ecological knowledge.

Although gender was not a significant predictor in the statistical analyses, the predominance of male participants (81.1%) should be considered when interpreting gender-related patterns of knowledge and is discussed further in the study limitations.

### Central taxa of wild edible plants

A total of **70 wild edible plant (WEP) species** belonging to **32 botanical families** were documented during the study. The complete inventory is presented in Table 2, which includes scientific names with author citations, voucher specimen codes, local Afaan Oromo names, edible plant parts, and detailed preparation methods together with associated traditional uses and local perceptions.

**Table 2:** Central taxa inventory of wild edible plants recorded in Aseko district

Voucher Code	Scientific Name	Family	Local Name	Edible Part(s)	Preparation/Processing & Uses/Perceptions
JSJ001	*Vachellia abyssinica* (Hochst. ex Benth.) Kyal. & Boatwr	Fabaceae	Lafto	Pods, seeds, gum	Pods roasted/boiled to soften; gum extracted and chewed. Critical famine food; highly valued for sustenance during dry seasons
JSJ002	*Senegalia mellifera* (Vahl) Seigler & Ebinger	Fabaceae	Qilxuu	Pods, seeds	Seeds roasted to reduce hardness. Famine food; consumed when cereals fail
JSJ003	*Vachellia robusta* (Burch.) Kyal. & Boatwr	Fabaceae	Laftoo Bosonaa	Pods, gum	Gum extracted and chewed; pods roasted. Famine food; gum important in dry seasons
JSJ004	*Vachellia seyal* (Del.) P.J.H.Hurter & Mabb	Fabaceae	Wachu	Gum, young pods	Gum extracted; young pods cooked. Famine food; major gum source
JSJ005	*Vachellia tortilis* (Forssk.) Galasso & Banfi	Fabaceae	Dadacha	Pods, seeds, gum	Pods roasted/boiled. Widely used famine food; highly resilient
JSJ006	*Anogeissus leiocarpa* (DC.) Guill. & Perr	Combretaceae	Birbirsa Gammoojjii	Gum	Gum extracted and chewed. Dryland gum source; famine food
JSJ007	*Balanites aegyptiaca* (L.) Delile	Balanitaceae	Beddeena	Fruit, seed oil	Fruit eaten raw; seeds cold-pressed for oil. High nutritional value; drought-tolerant
JSJ008	*Brucea antidysenterica* J.F. Mill	Simaroubaceae	Jiloolaafaa	Fruit	Small quantities consumed; traditional preparation essential. Dual-use: food + medicine; use with caution
JSJ009	*Calpurnia aurea* (Ait.) Benth	Fabaceae	Cekaa	Flowers	Flowers cooked thoroughly to reduce alkaloids. Traditional vegetable; requires careful processing
JSJ010	*Capparis tomentosa* Lam	Capparaceae	Harangamaa	Fruit, young shoots	Shoots cooked to reduce glucosinolates; fruit raw. Dual use; cook shoots to reduce compounds
JSJ011	*Carissa edulis* (Forssk.) Vahl	Apocynaceae	Agamsa	Fruit	Eaten raw. Highly preferred wild fruit; common snack; emerging local market value
JSJ012	*Carissa spinarum* L	Apocynaceae	Hagamsa	Fruit	Eaten raw. Common wild snack; highly accessible to children
JSJ013	*Clematis hirsuta* Perr	Ranunculaceae	Fiitii	Young shoots	Shoots cooked thoroughly to reduce protoanemonin toxin. Requires careful processing
JSJ014	*Clutia abyssinica* Jaub. & Spach	Euphorbiaceae	Ulee Foni	Young leaves	Leaves cooked; latex avoided (skin irritant). Rare; cook thoroughly
JSJ015	*Combretum molle* R.Br. ex G. Don	Combretaceae	Abaluu	Fruits (young)	Young fruits eaten raw. Seasonal fruit; highly valued
JSJ016	*Cordia africana* Lam	Boraginaceae	Wadeesa	Fruit	Eaten raw. Among top 3 most cited WEPs in Ethiopia; monitor local populations
JSJ017	*Cucumis ficifolius* A.Rich	Cucurbitaceae	Araddoo gurraatti	Fruit	Eaten raw or cooked. Cook if bitter to reduce cucurbitacins
JSJ018	*Dodonaea angustifolia* L.f	Sapindaceae	Iticha	Seeds	Seeds roasted thoroughly to reduce saponins. Traditional snack
JSJ019	*Dovyalis abyssinica* (A. Rich.) Warb	Salicaceae	Kooshimo	Fruit	Eaten raw. Sour edible fruit; highly cited in Ethiopian reviews
JSJ020	*Ehretia cymosa* Thonn	Boraginaceae	Ulaaga	Fruit	Eaten raw. Wild edible; seasonal availability
JSJ021	*Embelia schimperi* Vatke	Primulaceae	Haanquu	Fruit	Eaten raw. Wild edible fruit; dual medicinal use
JSJ022	*Erythrina brucei* Schweinf	Fabaceae	Walensuu	Flowers	Flowers cooked thoroughly to reduce alkaloids. Requires careful processing
JSJ023	*Euclea racemosa* Murr	Ebenaceae	Mi’eessaa	Fruit	Eaten raw. Drought-tolerant edible; dryland importance
JSJ024	*Faidherbia albida* (Delile) A. Chev	Fabaceae	Gerbi	Pods, seeds	Seeds roasted; pods boiled. Key agroforestry famine food
JSJ025	*Ficus sur* Forssk	Moraceae	Harbuu	Fruit (figs)	Eaten raw. Fig species; culturally significant
JSJ026	*Ficus sycomorus* L	Moraceae	Odaa	Fruit (figs)	Eaten raw. Cultural importance; sacred tree associations
JSJ027	*Ficus thonningii* Blume	Moraceae	Odaa Muka	Fruit (figs)	Eaten raw. Widely consumed; large figs
JSJ028	*Ficus vasta* Forssk	Moraceae	Qilxuu Guddicha	Fruit (figs)	Eaten raw. Important dryland fig; highly valued
JSJ029	*Flacourtia indica* (Burm.f.) Merr	Salicaceae	Hoca	Fruit	Eaten raw. Wild fruit; tart taste
JSJ030	*Gardenia ternifolia* Schumach. & Thonn	Rubiaceae	Danfaa	Fruit	Eaten raw. Edible; well-represented in Ethiopian WEPs
JSJ031	*Gardenia volkensii* K. Schum	Rubiaceae	Danfaa Bosonaa	Fruit	Eaten raw. Edible; wild relative of G. ternifolia
JSJ032	*Grewia bicolor* Juss	Malvaceae	Raafuu	Fruit	Eaten raw. Wild edible; mucilaginous texture
JSJ033	*Grewia ferruginea* Hochst. ex A. Rich	Malvaceae	Dokonu	Fruit	Eaten raw. Edible; common in midlands
JSJ034	*Grewia tembensis* Fresen	Malvaceae	Raafuu Dheeraa	Fruit	Eaten raw. Edible; regional importance
JSJ035	*Grewia villosa* Willd	Malvaceae	Raafuu Gurraacha	Fruit	Eaten raw. Important wild fruit; highly preferred
JSJ036	*Juniperus procera* Hochst. ex Endl	Cupressaceae	Hindesa	Berries	Used sparingly as flavoring; potent oils. IUCN NT; use in small quantities only
JSJ037	*Kigelia africana* (Lam.) Benth	Bignoniaceae	Kilimoo	Fruit (processed)	Fermented/roasted/brewed; never raw. Raw fruit causes irritation; traditional processing essential
JSJ038	*Lannea fruticosa* (Hochst. ex A. Rich.) Engl	Anacardiaceae	Buqqaatii Bosonaa	Fruit	Eaten raw. Traditional edible; wild relative
JSJ039	*Lannea schimperi* (A. Rich.) Engl	Anacardiaceae	Buqqaatii	Fruit	Eaten raw. Edible; sweet taste
JSJ040	*Lippia adoensis* Hochst. ex Walp	Verbenaceae	Sukaayee	Leaves	Infused as tea. Herbal tea; highly valued for digestion
JSJ041	*Maesa lanceolata* Forssk	Primulaceae	Abayi	Fruit	Eaten raw. Edible; small berries
JSJ042	*Ocimum gratissimum* L	Lamiaceae	Daamakasee	Leaves	Used fresh or dried. Condiment; spice
JSJ043	*Olea europaea L. subsp. Cuspidata*	Oleaceae	Ejersa	Fruit (olive)	Cured/brined; oil extracted. Wild olive; requires curing to remove bitterness
JSJ044	*Ormocarpum kirkii* S. Moore	Fabaceae	Hiddii Bosonaa	Seeds	Seeds roasted to reduce toxins. Traditional snack
JSJ045	*Pappea capensis* Eckl. & Zeyh	Sapindaceae	Qilxuu Daalacha	Fruit	Eaten raw. Nutritious; sweet
JSJ046	*Phoenix reclinata* Jacq	Arecaceae	Meexxi	Fruit (dates)	Eaten raw. Wild date; highly valued
JSJ047	*Podocarpus falcatus* (Thunb.) R.Br. ex Mirb	Podocarpaceae	Birbirsa	Aril only	Eat red aril only; discard toxic seed. IUCN NT; seed toxic; conservation priority
JSJ048	*Prunus africana* (Hook.f.) Kalkman	Rosaceae	Sukkeessaa	Fruit	Eaten raw; sustainable harvest essential. IUCN VU; high-value fruit
JSJ049	*Rhamnus prinoides* L’Her	Rhamnaceae	Geeshoo	Fruits	Fermented for traditional drinks (tella/tej). Beverage flavoring
JSJ050	*Rhus natalensis* Krauss	Anacardiaceae	Daboobessaa	Fruit	Eaten raw; washed first to remove urushiol traces. Popular seasonal fruit
JSJ051	*Searsia vulgaris* (Krauss) Moffett	Anacardiaceae	Summaa Diimaa	Fruit	Eaten raw. Similar to R. natalensis; tart
JSJ052	*Rubus apetalus* Poir	Rosaceae	Goraa Bosonaa	Fruit (berries)	Eaten raw. Berry; wild relative of blackberry
JSJ053	*Rubus steudneri* Schweinf	Rosaceae	Gora	Fruit (berries)	Eaten raw. Endemic Ethiopian blackberry; highly preferred
JSJ054	*Rumex nepalensis* Spreng	Polygonaceae	Shaabbee	Leaves	Leaves cooked; bitter water discarded. Contains oxalates; cook thoroughly
JSJ055	*Silene macrosolen* (A.Rich.) Rohrb	Caryophyllaceae	Waggarti	Young shoots/leaves	Young parts cooked thoroughly. Traditional green
JSJ056	*Solanum incanum* L	Solanaceae	Hidi orome	Fruit	Cooked thoroughly; ripe fruit only. Contains glycoalkaloids; traditional use only
JSJ057	*Solanum indicum* L	Solanaceae	Hiddii	Fruit, leaves	Cooked thoroughly; bitter water discarded. Bitterness indicates higher alkaloids
JSJ058	*Syzygium guineense* (Willd.) DC	Myrtaceae	Baddessa	Fruit	Eaten raw. Among top 3 most cited WEPs in Ethiopia; key WEP
JSJ059	*Teclea nobilis* Del	Rutaceae	Hadheessa	Fruit	Eaten raw. Woody climber fruit; tart
JSJ060	*Thymus schimperi* Ronniger	Lamiaceae	Xooshinee	Leaves	Used in tea/cooking. Tea/spice; highly valued
JSJ061	*Tragia cinerea* Pax	Euphorbiaceae	Laalessaa	Young leaves	Leaves cooked; latex avoided (irritant). Cook thoroughly
JSJ062	*Urtica simensis* Steud	Urticaceae	Doobbii	Leaves	Cooked to neutralize stinging hairs. Nutritious cooked green
JSJ063	*Urera hypselodendron* (Hochst. ex A. Rich.) Friis	Urticaceae	Haliilaa	Young leaves	Leaves cooked thoroughly to neutralize stinging hairs. Stinging climber
JSJ064	*Vachellia etbaica* (Schweinf.) Kyal. & Boatwr	Fabaceae	Waaccuu	Pods, gum	Gum extracted; pods processed. Acacia relative; famine food
JSJ065	*Vachellia nilotica* (L.) P.J.H.Hurter & Mabb	Fabaceae	Burquqqee	Pods, gum	Gum extracted; pods processed. Widely used gum source
JSJ066	*Verbascum sinaiticum* Benth	Scrophulariaceae	Gurra Harree	Young leaves	Young leaves cooked. Primarily medicinal; edible use limited
JSJ067	*Vernonia amygdalina* Del	Asteraceae	Ebicha	Young leaves	Leaves cooked thoroughly to reduce alkaloids. Bitter vegetable; widely used
JSJ068	*Ximenia americana* L	Olacaceae	Inkoyyee	Fruit, seeds	Fruit raw; seeds oil extracted. Nutritional; oil extraction
JSJ069	*Ziziphus mucronata* Willd	Rhamnaceae	Kurkuraa Bosonaa	Fruit	Eaten raw. Common; wild relative
JSJ070	*Ziziphus spina-christi* (L.) Desf	Rhamnaceae	Kurkuraa	Fruit	Eaten raw; dried for storage. Highly valued; emerging market pathways

The table presents the 70 documented wild edible plant species, including voucher specimen codes (JSJ001–JSJ070), accepted scientific names with author citations, botanical families, local Afaan Oromo names, edible plant parts, use categories, and detailed traditional preparation and processing methods. Indigenous food-processing practices used to improve palatability, reduce toxicity, and ensure safe consumption are also summarized

Taxonomic verification indicated a high level of confidence in species identification. Of the documented species, **52 (74.3%)** were classified as **fully verified**, whereas **18 species (25.7%)** were categorized as **verified with caution**, reflecting the requirement for specific traditional processing methods to reduce toxicity or improve edibility before consumption.

While Table 2 presents the central taxa inventory and indigenous preparation methods, the extended dataset including IUCN conservation statuses, source habitats, taxonomic verification notes, and detailed safety profiles for all 70 species is provided in Additional file 1.

**Fig. 2:**
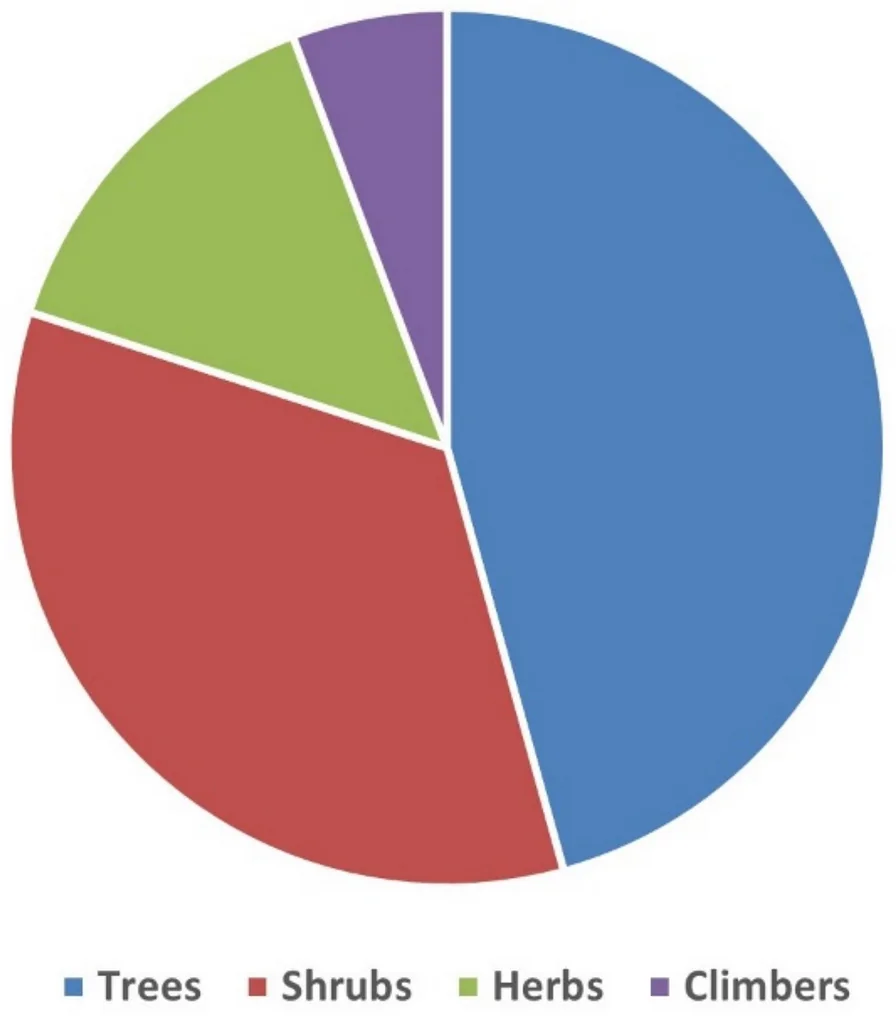
Growth forms of wild edible plants documented in Aseko district. Distribution of the 70 documented wild edible plant species according to growth form (trees, shrubs, herbs, and climbers)

Analysis of growth forms revealed that trees constituted the dominant category (45.7%, n = 32), followed by shrubs (34.3%, n = 24), herbs (14.3%, n = 10), and climbers (5.7%, n = 4) (Fig. 2). Most species (88.6%, n = 62) were harvested from wild habitats, whereas only 11.4% (n = 8) were associated with home gardens, cultivated fields, or managed landscapes.

### Use categories, edible parts, and preparation methods

**Fig. 3:**
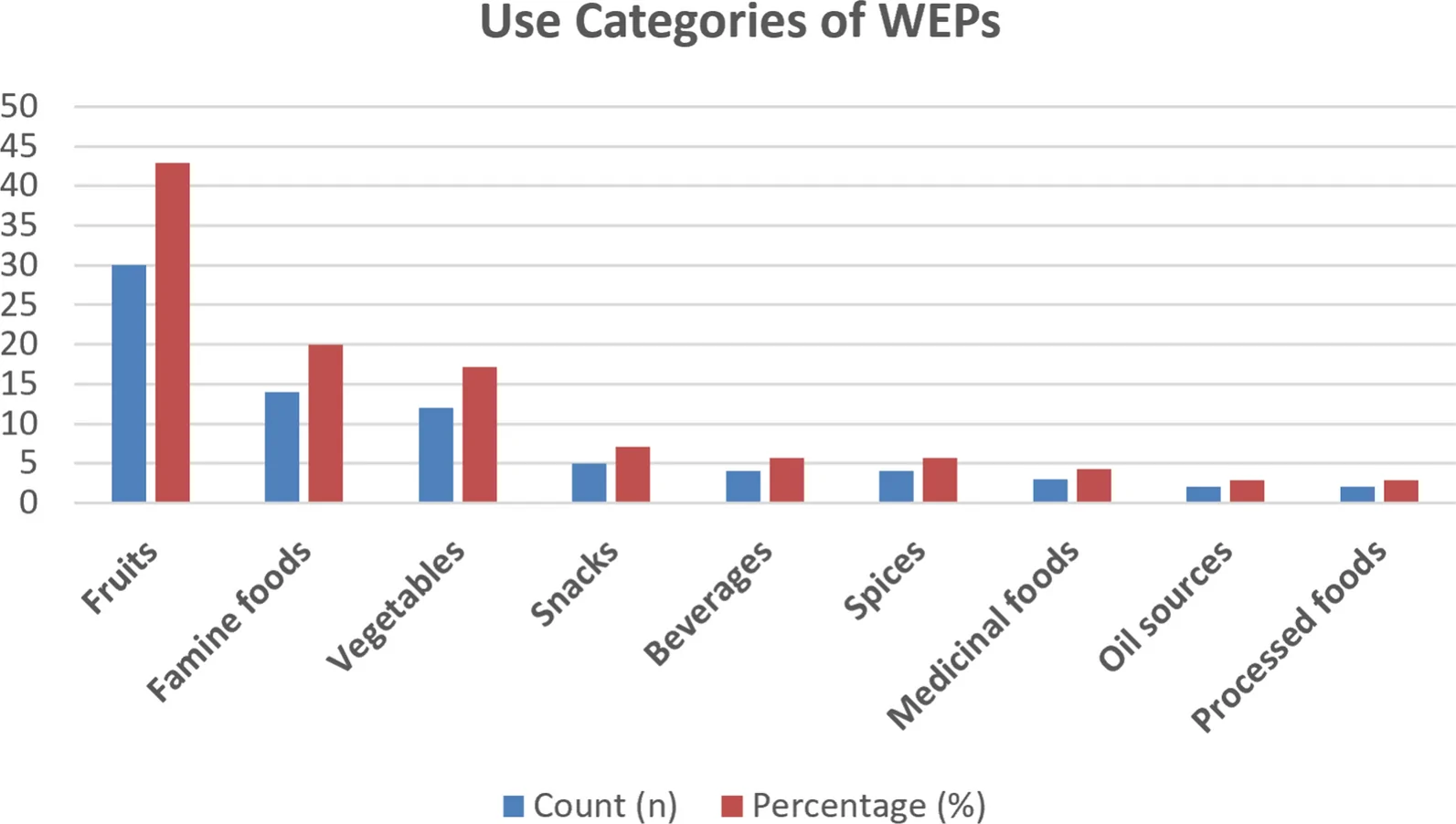
Use categories of wild edible plants documented in Aseko District. Distribution of the documented wild edible plant species according to their primary food-use categories

Wild edible plants were used for a wide range of food purposes, with **fruits** representing the dominant use category (**42.9%; n = 30**), followed by **famine-reserve foods** (**20.0%; n = 14**) (Fig. 3).

For the purposes of this study, **famine-reserve foods** were defined as plant species harvested and consumed primarily during periods of severe food shortage, including gums and hard-coated seeds. In contrast, **snacks** referred to species consumed casually in small quantities during routine daily activities.

**Fig. 4:**
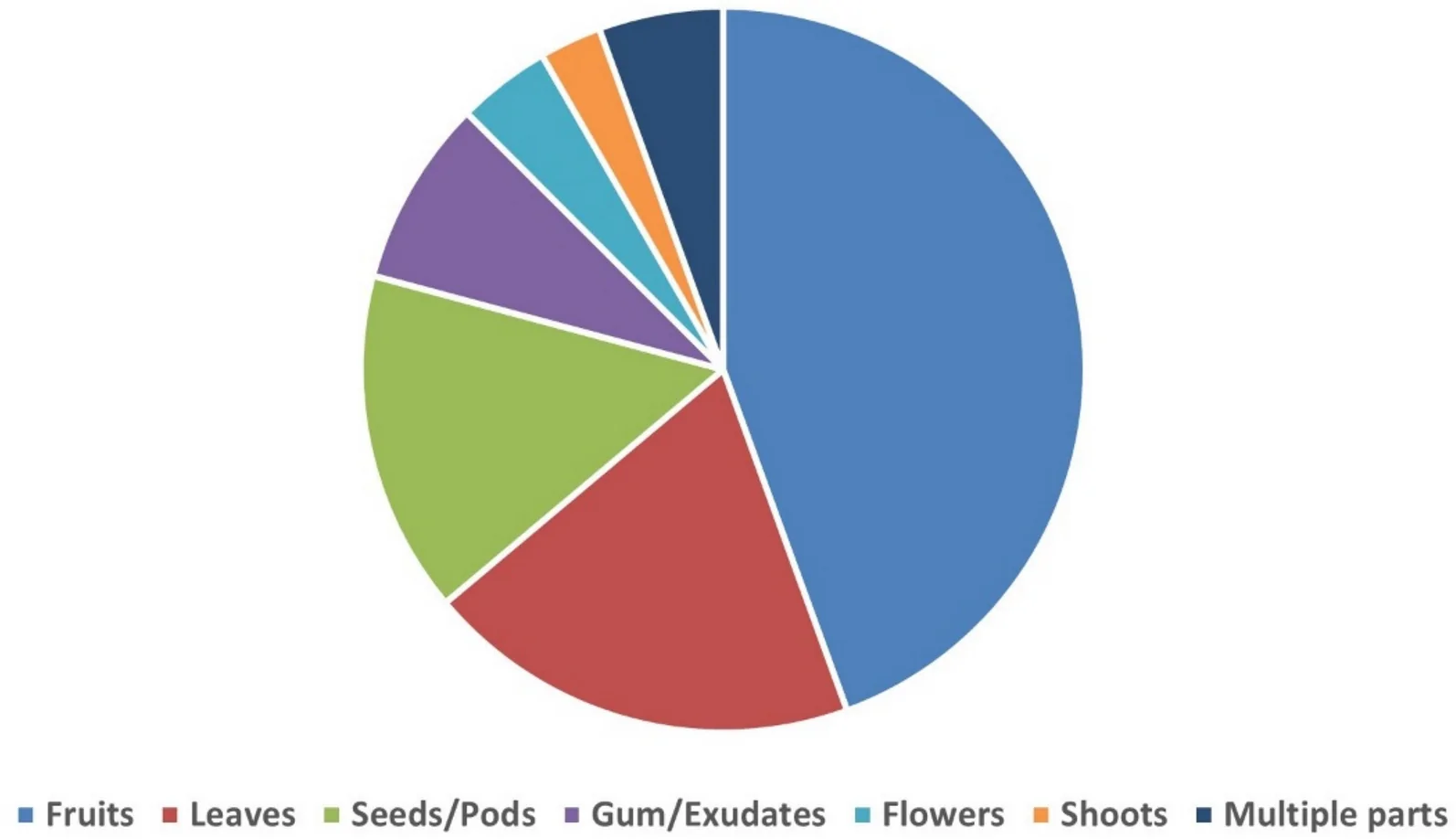
Edible plant parts utilized in Aseko district. Distribution of the edible plant parts harvested from the documented wild edible plant species

Regarding edible plant parts, **fruits** were the most frequently utilized (**45.7%; n = 32**), followed by other edible structures including leaves, seeds, flowers, pods, gums, and shoots (Fig. 4).

**Fig. 5:**
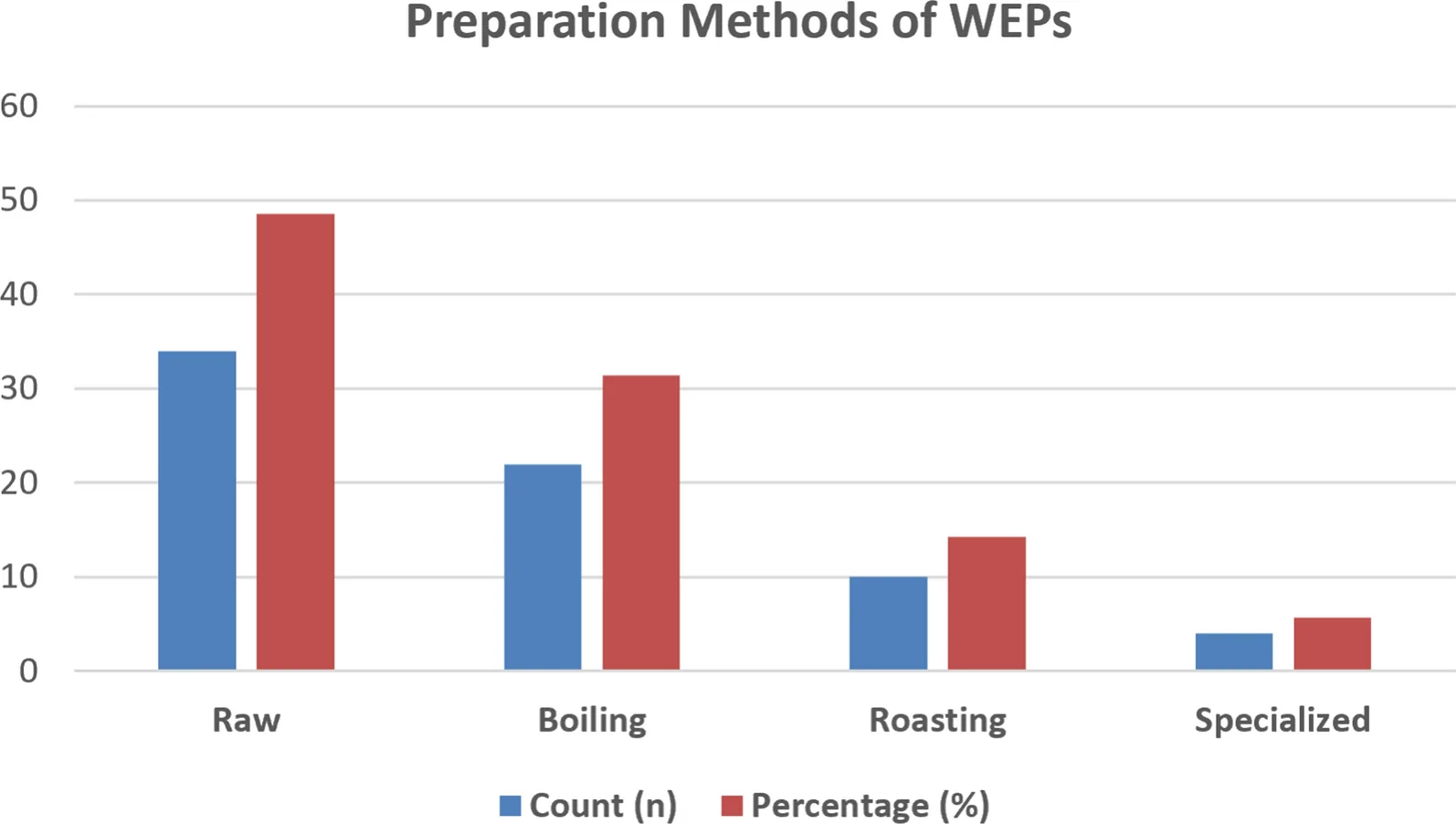
Traditional preparation methods of wild edible plants in Aseko District. Distribution of the principal traditional preparation and processing methods used before consumption of documented wild edible plant species

Preparation methods reflected well-developed indigenous food-processing practices designed to improve palatability and minimize health risks. Nearly half of the documented species (**48.6%**) were consumed raw, whereas **31.4%** required boiling, **14.3%** were roasted prior to consumption, and **5.7%** underwent specialized processing methods, including fermentation. Traditional safety knowledge associated with plant preparation and consumption was reported for **24 species (34.3%)**, highlighting the importance of indigenous knowledge in ensuring safe utilization (Fig. 5).

### Local perceptions of threats and conservation status

Informants identified **agricultural expansion** as the most serious threat to wild edible plant resources (**67.3%; n = 115**), followed by **fuelwood extraction** (**52.6%; n = 90**).

The conservation status of documented species was evaluated using the **IUCN Red List (Version 2025–1) **[45]**.**. Most species (**84.3%; n = 59**) were categorized as **Least Concern**, while **six species (8.6%)** were listed as **Near Threatened**, including *Juniperus procera*. Only **one species (1.4%)**, *Prunus africana*, was classified as **Vulnerable**. A complete species-by-species breakdown of IUCN Red List conservation statuses, source habitats, and local threat perceptions is available in Additional file 1.

The threat assessment presented here is based primarily on local informant perceptions. Future studies should integrate ecological field assessments and remote sensing approaches, including Sentinel-2 satellite imagery, to quantify land-use change, agricultural expansion, and grazing pressure affecting wild edible plant habitats.

**Fig. 6:**
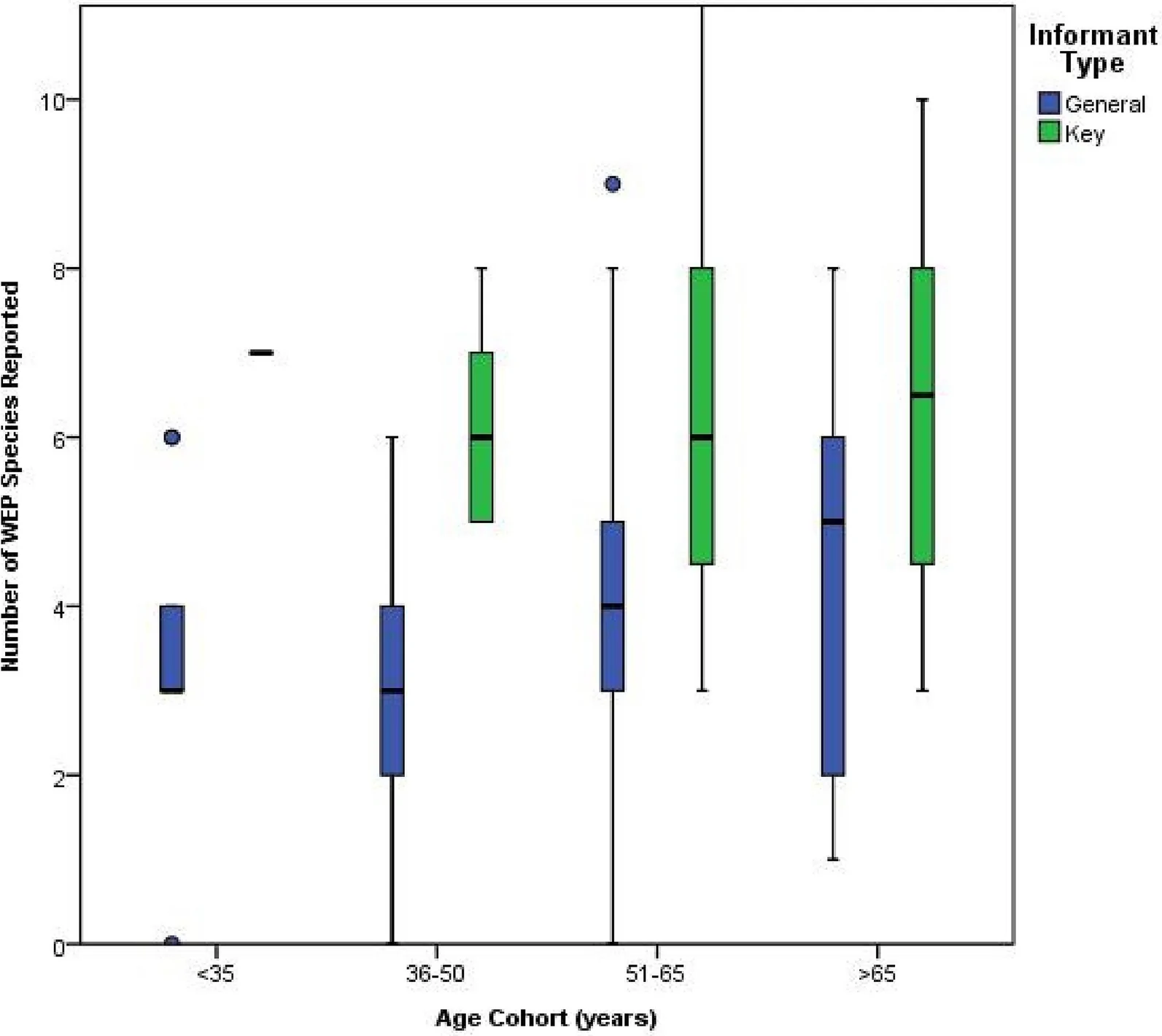
Intergenerational variation in wild edible plant knowledge. Box plots showing the number of wild edible plant species reported by informants according to age group and informant category. Older participants and key informants reported significantly more species than younger participants and general informants (Mann–Whitney U = 1245.5, *p* < 0.001)

Knowledge distribution exhibited clear intergenerational variation (Fig. 6). Older informants (> 50 years) reported significantly more species (mean ± SD: 4.8 ± 2.9) than younger informants aged < 35 years (2.1 ± 1.4), indicating progressive erosion of traditional ecological knowledge. Key informants consistently reported greater species richness and more detailed ethnobotanical knowledge than general informants within each age cohort, reflecting their specialized expertise and long-term experience. These findings suggest that wild edible plant knowledge in Aseko District is concentrated primarily among older community members and may be increasingly vulnerable to intergenerational decline as younger generations engage less frequently in traditional harvesting and food-processing practices.

### Cross-regional comparison and cultural interpretation

To address **Hypothesis 3 (H3)**, the wild edible plant (WEP) diversity documented in Aseko District was compared with previous ethnobotanical investigations conducted across different ecological and cultural regions of Ethiopia (Table 3). The **70 WEP species** recorded in the present study are comparable to inventories reported from the Afar Region (62 species) and southern Ethiopia (66 species), suggesting similar levels of floristic diversity despite differences in ecological conditions and livelihood systems. These similarities likely reflect the rich biodiversity of Ethiopian dryland and mixed farming landscapes as well as comparable ethnobotanical sampling approaches.

**Table 3:** Cross-regional comparison of wild edible plant studies in Ethiopia

Region/study	Ethnic group/ecology	Total species	Dominant family	Dominant edible part	Distinctive cultural or safety features
**Aseko (Present study)**	Oromo (Arsi)/Woodland	70	Fabaceae (15.7%)	Fruits (45.7%)	Famine-reserve gums, specialized fermentation, and detoxification protocols
South Ethiopia [4]	Derashe, Kucha, Gamo/Highland	66	Fabaceae	Fruits and leaves	High diversity of leafy vegetables and traditional processing practices
Afar Region [12]	Afar/Semi-arid pastoral	62	Fabaceae	Gums and fruits	Strong reliance on drought-resistant gum-producing species
Lower Omo [11]	Kara and Kwego/Riverine	36	Moraceae	Fruits	Riverine species and distinct semi-pastoral classifications

Bold text indicates data from the present study (Aseko District), whereas the remaining rows summarize findings from previously published studies.Comparison of the present study with selected ethnobotanical investigations conducted in different regions of Ethiopia. Variables compared include species richness, dominant botanical families, major use categories, principal edible plant parts, and notable cultural practices associated with the use and management of wild edible plants

The predominance of the **Fabaceae** family (**15.7%, n = 11**) is consistent with previous studies conducted in Ethiopian dryland and pastoral ecosystems [11, 12], reflecting the ecological adaptability, drought tolerance, and nutritional importance of numerous **Vachellia** and **Senegalia** species. Likewise, fruits represented both the dominant use category (42.9%) and the most frequently consumed edible plant part (45.7%). This pattern differs from several highland ethnobotanical studies, where leafy vegetables constitute the principal edible resource [4]. Such differences are most likely attributable to the woodland and savanna vegetation that characterizes Aseko District, where fruit-producing woody species are abundant.

From a cultural perspective, Oromo communities in Aseko maintain distinctive indigenous food-processing and safety practices. These include specialized fermentation techniques for *Kigelia africana* and carefully developed detoxification procedures for potentially toxic species, such as prolonged boiling of *Solanum incanum* to reduce glycoalkaloid concentrations and selective consumption of only the fleshy red aril of *Podocarpus falcatus*. These practices illustrate sophisticated indigenous food safety knowledge that enhances the safe utilization of wild edible plants. Comparable processing techniques were not documented in the selected ethnobotanical studies from the Afar pastoral communities or the Kara and Kwego communities of the Lower Omo Valley, suggesting that these practices represent locally developed cultural adaptations to the flora available within Aseko District.

**Quantitative Floristic Similarity and Shared Taxa**

To objectively evaluate floristic similarity and biocultural continuity, pairwise comparisons were conducted using the **Jaccard Similarity Index (J)** and the **Sørensen Similarity Index (S)** (Table 4). The analysis revealed moderate floristic similarity with neighboring Oromo communities, particularly **Guji Zone** (J = 0.191; S = 0.321) and **Mieso District** (J = 0.168; S = 0.288), indicating substantial overlap in traditionally utilized wild edible plant species. Intermediate similarity was observed with **Ensaro District** (J = 0.145; S = 0.252) and **Dibatie District** (J = 0.139; S = 0.244), whereas markedly lower similarity occurred with the semi-pastoral Kara and Kwego communities of the Lower Omo Valley (J = 0.043; S = 0.080). These differences likely reflect contrasting ecological conditions, vegetation types, and livelihood systems.

**Table 4:** Quantitative floristic similarity between Aseko district and selected Ethiopian ethnobotanical studies

Comparison study	Region/ethnic group	Total species (B)	Shared species (a)	Jaccard index (J)	Sørensen index (S)
Guji Zone [46]	Oromo (Southern Ethiopia)	36	11	0.191	0.321
Mieso District [47]	Oromo (Eastern Ethiopia)	41	12	0.168	0.288
Ensaro District [48]	Amhara (North Shewa)	43	10	0.145	0.252
Dibatie District [49]	Benishangul-Gumuz	54	12	0.139	0.244
Lower Omo [11]	Kara and Kwego (Semi-pastoralists)	36	3	0.043	0.080

Despite regional variation, several culturally important taxa—including *Carissa spinarum*, *Cordia africana*, *Syzygium guineense*, *Ziziphus spina-christi*, and species of *Ficus*—were consistently shared across Oromo-dominated regions, indicating the existence of a broadly conserved macro-regional ethnobotanical knowledge base. Nevertheless, the Aseko communities exhibited distinctive utilization of drought-resilient famine-reserve gum-producing species together with specialized fermentation and detoxification practices that were less frequently reported in adjacent midland and highland areas. These findings support the hypothesis that while core ethnobotanical knowledge is widely shared among Oromo communities, local ecological conditions have promoted the development of unique cultural adaptations in the selection, processing, and safe utilization of wild edible plants.

## Discussion

### Cross-regional comparison of wild edible plant diversity and floristic patterns

The present investigation documented 70 wild edible plant species, representing one of the most comprehensive inventories reported from southeastern Ethiopia. Comparison with previous Ethiopian ethnobotanical studies (Table 3) indicates that species richness recorded in Aseko District is comparable to or greater than that documented in several other regions, despite marked ecological and cultural differences. These variations are likely attributable to differences in vegetation type, rainfall regime, agroecological conditions, sampling intensity, and indigenous knowledge systems.

Our findings complement previous ethnobotanical investigations conducted in southeastern Ethiopia, indicating that although species composition varies among districts, indigenous knowledge systems consistently support dietary diversity, food security, and biodiversity conservation across Oromo communities [16]. Fabaceae was the dominant family, accounting for 15.7% (n = 11) of all documented species. Similar dominance of Fabaceae has been reported in several Ethiopian studies conducted in dry woodland and semi-arid ecosystems, reflecting the ecological success of nitrogen-fixing legumes under water-limited conditions. Species belonging to *Vachellia*, *Senegalia*, and related genera are particularly well adapted to drought-prone environments and provide edible fruits, seeds, gums, and other products that contribute substantially to household food security.

Fruits represented the most frequently consumed edible plant part (45.7%; n = 32), consistent with observations from many Ethiopian ethnobotanical studies where fleshy fruits constitute the principal category of wild edible foods. In contrast, investigations conducted in humid montane forests frequently report a greater contribution of leafy vegetables and tender shoots because of differences in floristic composition and climatic conditions. These regional differences demonstrate that wild edible plant utilization is strongly influenced by environmental gradients as well as by locally adapted cultural practices.

Beyond documenting species richness, the present study incorporated quantitative ethnobotanical indices, including Use Value (UV), Relative Frequency of Citation (RFC), Fidelity Level (FL), and Informant Consensus Factor (ICF), allowing for the objective evaluation of culturally important species and facilitating comparison with future ethnobotanical investigations. Such quantitative approaches strengthen the evidence base for identifying priority species for conservation, domestication, and sustainable utilization.

Quantitative floristic similarity analysis further demonstrated that Aseko shares a substantial proportion of its wild edible flora with neighboring Oromo communities. The relatively high Jaccard and Sørensen indices observed when comparing Aseko with the Guji Zone (*J* = 0.191; *S* = 0.321), Mieso District (*J* = 0.168; *S* = 0.288), and Ensaro District (*J* = 0.165; *S* = 0.283) indicate that culturally important species such as *Carissa spinarum*, *Cordia africana*, *Syzygium guineense*, and *Ficus* spp. are widely recognized across southeastern and central Ethiopia. This reflects shared linguistic heritage, cultural traditions, and overlapping dry Afromontane and woodland vegetation types. Conversely, the markedly lower similarity with the Kara and Kwego communities of the Lower Omo Valley (*J* = 0.082; *S* = 0.151) highlights how distinct ecological gradients, riverine flora, and semi-pastoralist livelihood systems drive localized ethnobotanical adaptations. Nevertheless, the shared utilization of core famine-reserve species across these diverse regions underscores the critical role of WEPs as a conserved biocultural safety net in Ethiopian food systems.

Wild edible plants classified as famine-reserve foods accounted for 20.0% (n = 14) of the documented species, highlighting their continued importance in supporting household food security during periods of drought, crop failure, and seasonal food shortages [50–52]. Rather than functioning solely as emergency foods, these species represent an integral component of indigenous livelihood strategies that enhance resilience to environmental and socioeconomic shocks [53]. Similar observations have been reported from other ethnobotanical studies in Ethiopia, where wild edible plants serve as safety-net resources during periods of food insecurity and climatic uncertainty [4, 12, 54, 55].

Several drought-tolerant species, including *Vachellia abyssinica*, *Vachellia tortilis*, *Senegalia mellifera*, and *Balanites aegyptiaca*, were reported to be harvested more frequently during years of poor agricultural production. Local communities have developed specialized preparation methods, including roasting, prolonged boiling, soaking, and mechanical extraction of edible gums, to improve palatability, reduce bitterness, and minimize potentially harmful compounds. These processing techniques demonstrate that indigenous knowledge extends beyond plant identification to include sophisticated methods for ensuring food safety and maximizing nutritional value.

The increasing frequency of climate variability in southeastern Ethiopia may further increase the importance of these drought-adapted species. Their continued availability provides an additional source of dietary diversity when cultivated crops fail, thereby strengthening community resilience against food insecurity. Consequently, wild edible plants should be recognized not merely as "fallback foods" but as strategic biological resources that contribute to climate-change adaptation, nutritional security, and sustainable rural livelihoods. From a food sovereignty perspective, the documented species also reflect the capacity of local communities to maintain culturally appropriate food systems based on indigenous knowledge, locally available biological resources, and traditional management practices. Protecting these resources therefore contributes simultaneously to biodiversity conservation, cultural heritage preservation, and sustainable development.

### Indigenous safety knowledge as cultural pharmacology

Approximately 34.3% (n = 24) of the documented wild edible plant species required specific preparation techniques before consumption, demonstrating the sophistication of indigenous knowledge systems developed to ensure food safety. Local communities employed a range of traditional processing methods, including boiling, roasting, soaking, fermentation, peeling, and selective harvesting of edible plant parts, to reduce bitterness, eliminate potentially toxic compounds, and improve palatability.

Representative examples include the prolonged boiling of *Solanum incanum* fruits to reduce glycoalkaloid toxicity, the fermentation or extended cooking of *Kigelia africana* fruits before consumption, and the selective consumption of only the fleshy red aril of *Podocarpus falcatus* while avoiding the toxic seed. These practices indicate that local knowledge encompasses not only species identification but also a detailed understanding of appropriate preparation methods and safe consumption.

The documented processing techniques closely correspond with the concept of cultural pharmacology, which recognizes that traditional food preparation methods are integral components of indigenous healthcare and food systems rather than simple culinary practices [56]. Such practices represent generations of empirical observation and experimentation, enabling communities to maximize nutritional benefits while minimizing health risks associated with naturally occurring plant secondary metabolites.

Recent ethnobotanical studies have similarly emphasized that local perceptions of plant safety, toxicity, and usefulness constitute an essential component of traditional ecological knowledge and should be considered when designing conservation, nutrition, and public health interventions [57]. Furthermore, integrating quantitative ethnobotanical approaches with qualitative documentation of indigenous processing knowledge provides a more comprehensive understanding of human–plant interactions across different cultural and ecological settings [58, 59]. The preservation of indigenous safety knowledge is therefore as important as conserving the plant resources themselves. As younger generations become increasingly disconnected from traditional food systems, the erosion of processing knowledge may reduce the safe utilization of wild edible plants even where the biological resources remain available. Documenting these culturally transmitted practices therefore contributes not only to ethnobotanical research but also to food security, nutrition, biodiversity conservation, and the safeguarding of intangible cultural heritage.

### Gendered knowledge domains and intergenerational knowledge erosion

The statistical analyses demonstrated that traditional ecological knowledge of wild edible plants differed significantly among demographic groups, supporting Hypothesis 1 (H1). Generalized Linear Model (GLM) analysis identified age as a significant positive predictor of species knowledge (IRR = 1.04, 95% CI: 1.02–1.06, *p* < 0.001), indicating that older participants reported a greater number of wild edible plant species than younger informants. Likewise, key informants possessed substantially greater ethnobotanical knowledge than general informants (IRR = 2.15, 95% CI: 1.80–2.50, *p* < 0.001), reflecting the value of accumulated experience and cultural specialization in maintaining traditional ecological knowledge.

The Mann–Whitney *U* test further confirmed significant intergenerational differences in species knowledge. Informants older than 50 years (median = 5 species, IQR = 3–7) reported significantly more wild edible plant species than participants younger than 35 years (median = 2 species, IQR = 1–3) (*U* = 1245.5, *p* < 0.001), with a large effect size (Cliff’s δ = 0.65). These findings indicate an ongoing decline in the transmission of ethnobotanical knowledge to younger generations, a pattern that has been documented in many traditional societies undergoing rapid socioeconomic and cultural change.

Although agroecological zone significantly influenced species knowledge (*p* = 0.032), with informants from lowland areas reporting greater species richness than those from midland and highland areas, gender was not a statistically significant predictor of knowledge in the fitted GLM (IRR = 1.12, 95% CI: 0.90–1.40, *p* = 0.210). Similarly, education level showed no significant association with species knowledge (*p* = 0.145). Post-hoc comparisons indicated that respondents from the lowland zone reported significantly more wild edible plant species than respondents from the midland and highland zones after Holm correction (adjusted p < 0.05), whereas no significant difference was detected between the midland and highland zones (adjusted p > 0.05).

Although we did not calculate the Combination Use Diversity Index (CUDI) due to the demographic limitations of our sample, the theoretical framework of CUDI [58] highlights the importance of measuring practical knowledge diversity across demographic cohorts. Our qualitative observations suggest that ethnobotanical knowledge is distributed across complementary gender-specific domains. Male informants reported broader familiarity with famine-reserve species and harvesting logistics, while female informants (though underrepresented in the sample) demonstrated specialized expertise in leafy vegetable preparation and fermentation. Applying cross-cultural analytical frameworks [59], these findings suggest that future studies with balanced demographic sampling should employ indices like CUDI to fully capture these complementary knowledge domains.

Nevertheless, the predominance of male participants (81.1%) may have influenced the breadth of knowledge documented. In many rural Ethiopian communities, responsibilities related to wild edible plants are distributed across gender-specific roles [60, 61]. Men are often more involved in fiesectld-based plant collection and resource management, whereas women frequently possess specialized knowledge of food preparation, processing, preservation, and household utilization [62–64]. Consequently, the lower participation of women may have resulted in the underrepresentation of female-specific knowledge domains, particularly those associated with leafy vegetables, processing techniques, and traditional culinary practices.

### Conservation implications and sustainable management of wild edible plants

The present study documented several anthropogenic and environmental threats affecting wild edible plant (WEP) resources in Aseko District, including agricultural expansion, fuelwood collection, overgrazing, charcoal production, and recurrent drought. Similar drivers of biodiversity loss have been widely reported in Ethiopian ethnobotanical studies and continue to threaten both plant diversity and the traditional ecological knowledge associated with these resources.

Community perceptions indicated that several culturally important species, including *Juniperus procera*, *Prunus africana*, and other multipurpose woody plants, have become increasingly scarce in their natural habitats. Because many of these species provide food, medicine, fuelwood, and construction materials simultaneously, harvesting pressure extends beyond their edible value alone. Consequently, conserving these species requires integrated landscape management rather than species-specific interventions.

The indigenous harvesting practices documented during this study demonstrate that local communities possess practical knowledge that contributes to the sustainable use of wild edible plants. Informants described selective harvesting of fruits, avoidance of unnecessary damage to parent plants, and protection of naturally regenerating individuals as customary management practices that support continued resource availability [65, 66]. Such locally developed management strategies represent valuable components of community-based conservation and should be recognized within formal biodiversity conservation programs [67–69].

The quantitative ethnobotanical indices generated in this study, particularly Use Value (UV) and Relative Frequency of Citation (RFC), provide an objective basis for identifying culturally important species that may warrant priority conservation and domestication. Species exhibiting high cultural importance but declining abundance should receive particular attention in restoration programs, community nurseries, and agroforestry initiatives. Strengthening collaboration among local communities, government agencies, research institutions, and conservation organizations will be essential for maintaining both wild edible plant diversity and the traditional ecological knowledge associated with these resources.

### Implications for conservation, sustainable utilization, and policy

The findings of this study have important implications for biodiversity conservation, food security, and sustainable rural development in Ethiopia. Species with high cultural importance, as identified through the quantitative ethnobotanical indices, should be prioritized for both in situ and ex situ conservation.

For the most threatened taxa identified in this study—specifically Juniperus procera (NT), Podocarpus falcatus (NT), and Prunus africana (VU)—I recommend strict in situ conservation through the expansion of community-managed exclosures and the enforcement of customary harvesting rules that prohibit the felling of mature trees. These strategies integrate seamlessly with local land-use practices by aligning with the protection of sacred groves (*Qallu* sites) and regulating access to communally owned forest patches. Conversely, for high-use, commercially emerging taxa such as *Ziziphus spina-christi*, *Carissa edulis*, and *Vachellia* spp., we recommend participatory domestication and integration into local agroforestry systems. Establishing community nurseries to propagate these species will reduce harvesting pressure on wild populations while providing sustainable livelihood opportunities. This domestication strategy integrates with local land-use practices by promoting the retention of multipurpose trees in agricultural landscapes and restoring degraded communal grazing areas.

The documented indigenous knowledge of harvesting, preparation, and safe utilization further demonstrates the importance of incorporating traditional ecological knowledge into agricultural extension services, biodiversity conservation programs, and local development planning. Community-based training, school environmental education, and intergenerational knowledge-sharing activities could contribute to preserving this knowledge while promoting sustainable resource management.

The increasing local commercialization of certain wild edible plant species may provide additional livelihood opportunities for rural households. However, sustainable harvesting guidelines, community monitoring, and appropriate local management institutions will be essential to prevent overexploitation of natural populations. Finally, continued collaboration among local communities, researchers, conservation organizations, and government institutions is necessary to ensure that future utilization of wild edible plant resources remains consistent with the principles of equitable benefit-sharing under the Nagoya Protocol and supports both biodiversity conservation and local livelihoods.

### Study limitations

Several limitations should be considered when interpreting the findings of this study. First, although the study included 206 informants distributed across six representative kebeles encompassing the major agroecological zones of Aseko District, the use of purposive and snowball sampling means that the findings may not be fully generalizable to all communities within the district or to other regions of Ethiopia.

Second, the predominance of male participants (81.1%) may have resulted in the underrepresentation of women’s traditional ecological knowledge, particularly regarding household food preparation, processing techniques, preservation methods, and the utilization of leafy vegetables. Future studies should adopt more gender-balanced sampling strategies to better capture complementary knowledge held by both men and women.

Third, although fieldwork was conducted over a one-year period covering both the *Belg* and *Kiremt* seasons, some ephemeral, rare, or highly localized wild edible plant species may not have been encountered during the survey period. Repeated long-term monitoring across multiple years would provide a more comprehensive assessment of seasonal variation in species availability and utilization.

Fourth, the study relied primarily on ethnobotanical interviews and field observations to document indigenous knowledge. While many traditional preparation methods correspond with published scientific evidence regarding food safety and toxicity reduction, laboratory analyses of phytochemical composition, nutritional value, and detoxification mechanisms were beyond the scope of the present investigation. Such analyses would further strengthen the scientific validation of indigenous food-processing practices.

Finally, conservation threats were evaluated primarily from community perceptions and field observations rather than through quantitative ecological assessments. Future research integrating vegetation surveys, population ecology, remote sensing, and land-use change analyses would provide a more comprehensive understanding of the conservation status of priority wild edible plant species and their habitats. Additionally, while the GLM accounted for broad agroecological zones, future studies should incorporate fine-scale ecological covariates, such as distance to forest edges and local forest cover percentage, to better isolate the environmental drivers of WEP knowledge distribution.

Despite these limitations, this study provides the first comprehensive and quantitatively supported documentation of wild edible plants used by Oromo communities in Aseko District. The integration of ethnobotanical inventories, quantitative indices, and statistical analyses contributes valuable baseline information for biodiversity conservation, food security, and the preservation of traditional ecological knowledge in southeastern Ethiopia.

## Conclusion

This study presents the first taxonomically verified and culturally contextualized ethnobotanical inventory of wild edible plants (WEPs) in Aseko District, documenting 70 species traditionally utilized by Oromo communities. By integrating rigorous taxonomic verification with quantitative ethnobotanical indices, the research provides a robust baseline for understanding the region’s biocultural diversity and traditional ecological knowledge.

The findings demonstrate that traditional food systems encompass sophisticated plant identification, processing, and safety practices—such as specialized fermentation and detoxification—that function as culturally evolved mechanisms for minimizing dietary risks. This highlights the depth and practical relevance of local ecological knowledge in ensuring food safety and maximizing nutritional value.

Furthermore, the study reveals both the resilience and the vulnerability of these traditional knowledge systems. While customary conservation practices continue to support the sustainable management of WEP resources, significant intergenerational knowledge erosion and gender-specific knowledge domains underscore the urgent need for targeted conservation and knowledge transmission efforts.

Ultimately, this research positions wild edible plants not merely as fallback foods, but as culturally embedded, ecologically adaptive, and nutritionally vital resources. They contribute meaningfully to biodiversity conservation, community-based food security, climate change adaptation, and sustainable rural livelihoods in southeastern Ethiopia. These findings provide a critical scientific foundation for integrating WEPs into district-level development planning, participatory conservation initiatives, and culturally appropriate strategies for strengthening food sovereignty and biocultural resilience.

## Supplementary Information

Additional file 1: Title: Extended ethnobotanical and ecological dataset of wild edible plants in Aseko District. Description: This Excel file contains the comprehensive inventory of the 70 documented wild edible plant species. It includes standardized voucher codes, accepted scientific names with author citations, botanical families, local Afaan Oromo names, edible parts, use categories, source habitats, IUCN Red List conservation statuses, traditional preparation methods, safety notes, and taxonomic verification documentation against POWO/WFO

## Data Availability

The complete species inventory dataset supporting the conclusions of this study is included within the article and its supplementary material (Additional file 1: Additional_file 1.xlsx). Ethnobotanical interview data and informant demographic records are available from the corresponding author upon reasonable request, subject to ethical restrictions related to community knowledge sovereignty and participant confidentiality.
